# Association Between Health‐Related Physical Fitness and Cognition in Preschoolers: MOVI‐HIIT Study

**DOI:** 10.1111/sms.70268

**Published:** 2026-03-26

**Authors:** María E. Visier‐Alfonso, Mairena Sánchez‐López, Bruno Bizzozero‐Peroni, Ana Díez‐Fernández, Abel Ruiz‐Hermosa, Vicente Martínez‐Vizcaíno

**Affiliations:** ^1^ Universidad de Castilla‐La Mancha, Faculty of Nursing Cuenca Spain; ^2^ Universidad de Castilla‐La Mancha, Health and Social Research Center Cuenca Spain; ^3^ Universidad de Castilla‐La Mancha, School of Education Ciudad Real Spain; ^4^ Universidad de la República, Department of Physical Education and Health, Higher Institute of Physical Education Rivera Uruguay; ^5^ Universidad de Extremadura, Faculty of Sport Sciences, ACAFYDE Cáceres Spain; ^6^ University of Huelva, Faculty of Education, Sports Sciences, and Psychology Huelva Spain; ^7^ Universidad Autónoma de Chile, Faculty of Medicine Talca Chile

**Keywords:** cognition, executive function, health‐related physical fitness, preschool

## Abstract

Preschoolers with better health‐related physical fitness (HRPF) have better cognitive and brain functioning. This study examined the associations between health‐related physical fitness and cognitive domains in preschool children, including the independent role of fitness components, potential moderators, and links to low cognitive achievement. This was a cross‐sectional study analyzing baseline data from a randomized controlled trial (MOVI‐HIIT) including 522 preschoolers aged 3–5 years from 9 schools in Ciudad Real, Spain. Speed‐agility, upper and lower body muscle strength, cardiorespiratory fitness (CRF), and balance were measured with the PREFIT battery. Cognition was measured using the Differential and General Aptitude Battery (numerical concepts and vocabulary), Flanker Task (inhibition), Dimensional Change Card Sort (cognitive flexibility), and Span of words (working memory); sex, age, socioeconomic status, and screen time were measured as covariates. All HRPF components were positively associated with all cognitive domains (*r* = 0.11–0.38; all *p* ≤ 0.050). ANCOVA models showed that children in higher categories of fitness components, except for lower body strength, had significantly better scores in numerical concepts, vocabulary, inhibition, and working memory. Cognitive flexibility was only associated with balance. Logistic regression models revealed that high levels of speed‐agility, upper body strength, CRF, and balance were associated with reduced odds of low cognitive performance. HRPF was associated with cognitive performance in preschoolers, with variations by fitness and cognitive domains. Balance, CRF, and speed‐agility emerged as key components associated with better cognitive outcomes. These findings support the relevance of HRPF in early cognitive development.

**Clinical Trial Registration (If Any):** Mairena Sánchez‐López NCT04863040. Deidentified individual participant data (including data dictionaries) will be made available, in addition to study protocols, the statistical analysis plan, and the informed consent form. The data will be made available upon publication to researchers who provide a methodologically sound proposal for use in achieving the goals of the approved proposal. Proposals should be submitted to mariaeugenia.visier@uclm.es

**Abbreviations**
BADyG‐I: Assessment Battery for Early Childhood Education students
BMI: body mass index
CRF: cardiorespiratory fitness
DCCS: Dimensional Change Card Sort
FT: Flanker Task
HRPF: health‐related physical fitness
SES: socioeconomic status

## 1 Introduction

Cognitive development during early childhood is a critical determinant of later academic achievement, mental health, and social functioning [1, 2]. The preschool years represent a sensitive period for the maturation of core cognitive domains, including executive functions, language, and reasoning, which lay the foundation for school readiness and lifelong learning [3, 4]. Environmental and behavioral factors acting during this stage may have lasting effects on neurodevelopment, highlighting the importance of identifying modifiable determinants of cognitive health early in life [5].

Health‐related physical fitness (HRPF) in children is linked with physical and mental health [6], and cognitive development [7]. However, several components of HRPF, particularly cardiorespiratory fitness, muscular strength, motor competence, and flexibility, have shown declining trends in children over recent decades [8, 9], largely driven by increasing sedentary behaviors, such as screen time, and unhealthy lifestyle patterns [10, 11]. Although evidence on temporal trends in preschoolers is more limited, these patterns could be especially concerning during the preschool years, a critical window for both motor and cognitive development [12]. Evidence suggests a positive association between HRPF and cognitive performance in school‐aged children [13] with links to executive functions [14] potentially mediated by neurobiological, behavioral, and psychosocial mechanisms [15]. However, research in preschool populations remains limited and inconsistent [16], often constrained by small samples and methodological variability [12]. In this context, the PREFIT battery has emerged as a feasible and reliable tool to comprehensively assess HRPF in preschool children [6, 17], enabling large‐scale and methodologically robust research in this age group.

Evidence suggests a positive association between HRPF and cognitive performance in preschool children [13], with particularly consistent links to executive functions such as inhibitory control and working memory [16, 18]. These associations are thought to be mediated by neurobiological, behavioral, and psychosocial mechanisms, including increased cerebral blood flow, neural plasticity, motor learning, and attentional engagement [15]. However, research in preschool populations remains limited and inconsistent, often constrained by small sample sizes, heterogeneous cognitive assessments, and insufficient control for key confounders such as socioeconomic status, screen time, and body composition [19].

From a developmental perspective, different components of HRPF may relate to specific cognitive domains through partially distinct mechanisms. Cardiorespiratory fitness has been associated with neurobiological processes such as increased cerebral blood flow and neural plasticity [20], which are thought to preferentially support executive functions in children, particularly inhibitory control and working memory [21]. Motor‐related components, including speed–agility and balance, involve complex coordination, sensorimotor integration, and attentional control, which may be especially relevant for cognitive regulation processes in early childhood [21, 22]. Muscular strength may reflect broader neuromuscular maturation and overall physical activity exposure, potentially contributing to cognitive development [23].

Importantly, during preschool years, cognitive domains remain weakly differentiated and better represented by a largely unitary construct [24, 25], suggesting that mechanisms linking HRPF and cognition may extend beyond executive functions to broader abilities such as reasoning and vocabulary [26]. These domains are closely related to school readiness and later academic achievement yet remain underexplored in relation to HRPF in early childhood. The present study was designed to provide a comprehensive examination of multiple HRPF components and cognitive domains in preschool children. While informed by existing theoretical and empirical evidence, the analyses were partly exploratory, aiming to clarify domain‐specific patterns that remain insufficiently studied in early childhood. Clarifying which health‐related physical fitness (HRPF) components are most strongly associated with executive functions and broader cognitive domains may help inform early childhood health and educational strategies. Accordingly, this study aimed to examine the associations between HRPF and multiple cognitive domains in preschool children and to disentangle the specific contributions of individual HRPF components. We further investigated whether these associations were moderated by age, sex, socioeconomic status, and screen time. As a secondary objective, we aimed to identify HRPF components associated with low cognitive achievement.

## 2 Materials and Methods

This was a cross‐sectional analysis of data from baseline measurements of a cluster‐randomized trial (trial registration: NCT04863040) designed to test the effectiveness of an active breaks intervention (MOVI‐HIIT) in children 3–6. Baseline assessments were conducted after cluster randomization but prior to intervention implementation. The Clinical Research Ethics Committee of the General Hospital of Ciudad Real (Spain) approved the study protocol (REG:C‐254), which was also approved by the director and board of governors of each school. A letter was sent to the families requesting written consent for the child's participation in the study. Additionally, children were asked for verbal consent prior to participating in each test.

All children in the second and third courses of preschool education from the nine schools in urban and rural locations involved in the study were invited to participate (*n* = 622). Parents/guardians provided written consent for 522 children (response rate = 83.9%). The final analytic sample included 522 preschoolers (51.5% girls, age range 45–75 months, mean age 57 months [SD = 7]), who met the following inclusion criteria: (1) enrolled in the second or third course of preschool education; (2) understanding of Spanish language; (3) not having any learning disability; (4) not having any type of physical or mental disorder that parents and/or teachers had identified; (5) having the collaboration of a family member to answer questionnaires about free‐time family habits; and (6) consent of the parent or guardian for participating in the study.

The study variables were measured at school by trained investigators under standardized conditions during September–October 2022.

*Health Related physical fitness* was measured using the PREFIT battery [6]. All tests were administered according to standardized PREFIT procedures; feasibility and test–retest reliability for each test in preschool children have been previously reported [17]. All tests were conducted in the school gymnasium on the same day and during the last three hours of the morning. Before testing, participants completed a standardized 3‐min warm‐up consisting of a brief game involving running and jumping. Tests were administered individually in a randomized order, except for the cardiorespiratory fitness test, which was consistently performed last and carried out in groups of eight children. Throughout the assessment, evaluators provided continuous standardized encouragement to maximize participants' effort and performance. The administration procedures for each test are described below:

*Speed‐Agility*, using the 4 × 10 m shuttle run test. The lines were marked on the floor using yellow adhesive tape. Children run four times between two lines (10 m apart) as fast as possible. Two evaluators were stationed at each end, and participants were required to touch the evaluator's hand (held just beyond the line) and return as quickly as possible. Two attempts were made with an interval of 5 min, and the best time (seconds) was used for analysis. Less time represents better results.

*Lower Body Muscle Strength*, Using the Standing Broad Jump Test. From a starting position immediately behind a line, with feet shoulder‐width apart, the preschoolers jump horizontally to achieve maximum distance on a non‐slip mat featuring a marked take‐off line and 5‐cm graduations. The best of three attempts was recorded (cm).

*Upper Body Muscle Strength*, Using an Analogue Version of Dynamometer With Adjustable Grip TKK 5401 Grip‐DW (Takeya, Tokyo, Japan). The grip span was fixed at 4.0 cm. Preschoolers applied a gradual, continuous maximal squeeze for ≥ 2–3 s, performing two alternating trials with each hand, with the elbow fully extended and no other body contact with the dynamometer; the mean (kg) of the four measurements was calculated.

*CRF*, using the adapted and validated version PREFIT of the Course Navette test (20‐min shuttle run test) for preschoolers [27]. Children were asked to run between two lines 20 m apart while keeping pace with audio signals. The initial speed was 6.5 kmh‐1, which was increased by 0.5 kmh‐1. In each group of eight children, two evaluators ran ahead to help maintain the required pace. The number of laps completed was recorded.

*Balance*, Using the One‐Leg Stance Test. The child stood quietly on one foot with the contralateral leg flexed at the knee, maintaining the position for as long as possible. Two attempts were made with each leg, and the average time (seconds) was recorded.

*Cognition* was assessed in quiet conditions using the following tests:

*‐Intelligence and Mental ability*, with two dimensions of the Differential and General Aptitude Assessment Battery for Early Childhood Education students (BADyG‐I) for preschool children [28], which has been developed and adapted to preschool sample, showing good‐to‐excellent reliability for composite scores (α = 0.82–0.92) and acceptable construct validity supported by internal‐structure analyses in large normative samples. In both tests, the final score was computed as the sum of correct responses, with each correct answer awarded one point, yielding a maximum possible score of 18.

*Verbal understanding of basic quantitative/numerical concepts*, with the scale *quantitative/numerical concepts* that assess knowledge and understanding of basic concepts of quantity and number, essential for numerical reasoning. The participants were asked to choose the drawing that fulfills the term presented (counting, addition or subtraction, equating quantities and/or dimensions, discriminating numbers, or arithmetical symbols).

*Vocabulary or assimilation of basic knowledge of the environment* was measured with the scale *Information*. This test assessed the assimilation of data related to the socio‐cultural environment, memorized knowledge, and knowledge assimilated through oral exchange. The child was required to select the drawing that meets the definition expressed by the evaluator.

*Inhibition* and *cognitive flexibility* were measured using the NIH Toolbox [29] which was specifically designed and validated for children aged 3–15 years. Psychometric analyses showed adequate to excellent test–retest reliability (intraclass correlation coefficients 0.92), as well as satisfactory convergent and discriminant validity with well‐established developmental instruments [30]. We used the validated Spanish version, which has shown high test–retest reliability (0.59–0.69) and convergent validity [31]. *Inhibition* was measured an adapted version of the Eriksen Flanker Task [32]. Children were required to indicate the left–right orientation of a centrally presented stimulus while inhibiting attention to the potentially incongruent stimuli surrounding it. In some trials, the orientation of the flanking stimuli is congruent with the orientation of the central stimulus, and in other trials, incongruent. The task included four practice trials, and if passed, a 20‐trial block was presented. The trials consisted of a sequence of congruent and incongruent sets of arrows. Using a two‐vector method that incorporated both accuracy and reaction time, a final score was calculated for participants who maintained a high level of accuracy (> 80%) as follows: (0.25 × number of correct responses) + 5 –LOG10 [(congruent reaction time + incongruent reaction time/2)]. For children scoring < 80%, a total score considering accuracy was calculated. Unadjusted computed scores were used for the analysis [33]. *Cognitive flexibility* was measured using the Dimensional Change Card Sort test [32]. This test presented a stimulus by “color” or “shape,” and participants were asked to adapt their response according to the relevant dimension. The task included four practice trials; if passed, children were presented a 30‐trial block with both “shape” and “color” requirements. With accuracy percentage and reaction time on pre‐switch and post‐switch, a raw score was obtained, using a two‐vector method that incorporated both accuracy and reaction time, a final score was calculated for participants who maintained a high level of accuracy (> 80%) as follows: (0.167 × number of correct responses) + 5 –LOG10 [(congruent reaction time + incongruent reaction time/2)]. For children scoring < 80%, a total score considering accuracy was performed. Unadjusted computed scores were used for the analysis [33].

*Working memory* was measured with the Word Span [34], based on the Digit Span subtest from the Wechsler Intelligence Test for Children‐3rd edition (WISC‐III) [35]. According to Thorell and Wählstedt [34] the preschool version uses words rather than digits due to the age of the participants. Using the scores from 24 children between the ages of 4–5 years tested two weeks apart, the test showed adequate test–retest reliability, 0.67. The Spanish adaptation of this test was used, in this version, the words were selected from among familiar concepts and adapted to the development of preschool vocabulary (e.g., cat, tree, and milk) [36, 37]. Word Span includes two tasks: Word Span Forward, and Word Span Backward. In the Word Span Forward task, the participant is asked to repeat a sequence of words of increasing length from memory in the same order. The length of the sequence varies between two and seven words, and two different sequences are presented for each length. The Word Span Backward task has the same characteristics as the previous one, but the process is different as the participant is asked to repeat the words in the reverse order from the spoken sequence. This task finishes when the participant is unable to remember either of the two same‐length sequences. In each task, a point is scored for each correctly remembered sequence. In studies with older samples, Word Span Forward has been considered a measure of processes of short‐term maintenance of information, and Word Span Backwards a measure more related to working memory capacity [38]. However, in individuals with low memory capacity (e.g., preschoolers), effortful processing is needed even in relatively simple tasks [34]. Consequently, we decided to include WSF as a measure of executive function as in previous studies [36, 37]. A mean final score is calculated [34].

Although executive function is commonly operationalized into inhibitory control, working memory, and cognitive flexibility, evidence suggests that in early childhood these components may be weakly differentiated and better represented by a largely unitary construct [24, 25]. In the present study, these domains were analyzed separately based on the structure of the assessment instruments and prior task‐based research in preschool samples [13, 18]. However, this operational distinction does not necessarily imply fully independent latent constructs at this developmental stage. No substantial floor or ceiling effects were observed, and no participants were excluded based on task performance.

*Weight* was measured with the average of two measurements (Seca861; seca GmbH & Co. KG, Hamburg, Germany scale), with a measurement resolution of 0.1 kg. Children were measured barefoot and in light clothing. *Height* was measured with the average of two measurements with a wall‐mounted measuring rod (Seca222; seca GmbH & Co. KG, Hamburg, Germany), to the nearest 0.1 cm, with the participant standing upright. *Body Mass Index (BMI)* was calculated using the formula weight (kg)/height (m^2^). *Waist circumference* was measured with the average of three measurements of waist circumference with a flexible, non‐stretch (non‐elastic) tailor measuring tape (commercially available; manufacturer not indicated on the device), at the midpoint between the last rib and the iliac crest, at the end of normal expiration, and recorded to the nearest 0.1 cm. *Body fat* was calculated with the mean of two measurements by 8‐electrode electrical bioimpedance model Tanita BC‐418 MA (Tanita Corp., Tokio, Japan), with results displayed and recorded with a resolution of 0.1% for body fat percentage.

*Socioeconomic status (SES)* was assessed by a questionnaire completed by parents based on the scale proposed by the Spanish Society of Epidemiology [39]. The questionnaire asks the level of education and occupation of both parents. Three SES categories were obtained using both the parents' education and occupation: lower, middle, and high. A higher score indicated higher SES.

*Physical activity* and s*creen time* were assessed by ad hoc questionnaire completed by parents, in which they were asked about the number of hours that the child spent in physical activities and the number of hours of leisure time that the child spent viewing TV or using other electronic devices (computers, tablets, smartphones, or videogames) during the weekdays and weekend. The average of h/day of physical activity and screen time was calculated.

*Age* was calculated as the difference between the date of assessment and the date of birth and expressed in months as a continuous variable, to capture fine‐grained developmental differences during early childhood.

### 2.1 Statistical Analysis

To examine the normal distribution of continuous variables, graphical, and statistical (Kolmogorov–Smirnov test) methods were assessed. All continuous variables fit well within a normal distribution. Exploration of outlier and missing values (less than 10%) was performed. Outliers were examined visually and statistically; those identified as extreme but valid values were retained, while implausible or data‐entry errors were excluded from the analyses. Means (SDs) and percentages were calculated. *T*‐test and chi‐squared tests for independent samples was used to test for sex differences. In addition to *p*‐values, effect sizes were reported (Cohen's d for *t*‐tests and Cramer's V for chi‐squared tests). Effect sizes were calculated using Cohen's d and interpreted as small (0.20), medium (0.50), and large (0.80) and Cramer's V for SES interpreted as small (0.10), medium (0.30), and large (0.50). To examine the association between HRPF variables, cognition variables, and potential confounders, bivariate correlation coefficients were estimated (Pearson's r for approximately normally distributed variables and Spearman's ρ otherwise), reporting 95% CIs for correlation coefficients. The speed/agility variable was inverted. Categories of each HRPF component were calculated following the same procedure as previously reported elsewhere [16]. The categories for speed‐agility, lower body muscle strength, upper body muscle strength, CRF and balance correspond to the first quartile (Low), second and third quartiles (Middle), and the fourth quartile (High). Specifically. Low: > 18.4 seg in speed‐agility, < 56 cm in lower body muscle strength, < 5 kg in upper body muscle strength, < 10 laps in CRF, and < 8 seg in balance; Middle: 18.4–16.7 seg in speed‐agility, 57–85 cm in lower body muscle strength, 5.75–7.75 kg in upper body muscle strength, 11–23 laps in CRF, and 8–22 seg in balance; and High: > 15.6 seg in speed‐agility, > 86 cm in lower body muscle strength, > 8 kg in upper body muscle strength, > 24 laps in CRF, and > 22 seg in balance. Then, analysis of covariance (ANCOVA) was used to test differences in mean cognitive scores across categories of HRPF, with adjustments for age, sex, SES, and screen time, variables without significant correlations were excluded as covariates. For ANCOVA models, we report the main effect of HRPF category together with partial eta‐squared (ηp^2^) as an effect size and adjusted marginal means with pairwise differences. Post hoc pairwise comparisons were performed using the Bonferroni correction for multiple testing.

Additionally, we estimated a structural equation model to examine the association between physical fitness and cognition at the latent level. Two latent variables were specified: physical fitness and executive functions. To evaluate whether this association varied across individual and contextual characteristics, moderation effects were tested for age, sex, SES, and screen time. Latent interaction terms were constructed using the product‐indicator approach with mean‐centered indicators. All moderators were also included as main effects in the structural model. Models were estimated using robust maximum likelihood (MLR) with full information maximum likelihood to handle missing data. Model fit was evaluated using the χ^2^ test, CFI, TLI, RMSEA, and SRMR.

Finally, to examine the odds of low cognitive achievement, logistic regression models were performed. For logistic regression analyses, cognitive outcomes were dichotomized to define low achievement, and fitness variables were categorized into two categories as in previous studies [16] to facilitate interpretation and risk estimates. Low cognitive achievement was defined as the lowest decile (*p* < 10). Then, logistic regression models with each HRPF category as the predictor (low levels as the reference) to estimate the odds of low cognitive achievement in each cognitive domain, adjusting for confounders.

All analyses were conducted using the software R (v.4.4.3) and RStudio (v.RStudio 2024.12.1 + 563), using the lavaan package for structural equation models. The level of significance was set at *p* ≤ 0.050.

## 3 Results

The sample characteristics are summarized in Table 1. Regarding HRPF, boys showed higher performance in speed–agility, lower body strength, upper body strength, and CRF, whereas girls performed better in balance (all *p* < 0.01). However, effect sizes for these differences ranged from small to small‐to‐moderate (Cohen's d = 0.24–0.44). No statistically significant differences were found between boys and girls for any cognitive variable, and all corresponding effect sizes were trivial (Cohen's d ≤ 0.10).

**TABLE 1:** Characteristics of the sample.

Variables	Total	Boys	Girls	*p*	*Cohen's d*
	M (SD)	M (SD)	M (SD)		
	*n* = 522	*n* = 253	*n* = 269		
Age (months)	57 (7)	57 (8)	57 (6)	0.301	*0.03*
Height (cm)	108.1 (5.7)	108.9 (5.9)	107.3 (5.5)	**0.001**	*0.28*
Weight (kg)	18.2 (3.5)	18.6 (3.6)	17.9 (3.5)	**0.012**	*0.21*
BMI (kg/m^2^)	15.5 (2.0)	15.6 (2.0)	15.4 (2.1)	0.206	*0.08*
Waist circumference (cm)	53.3 (5.7)	53.6 (5.3)	53.1 (6)	0.317	*0.10*
Body fat (%)	20.4 (4.8)	20.6 (4.2)	20.3 (5.3)	0.142	*0.04*
SES					
Low	91 (22)	43 (20)	48 (23)		
Middle	233 (55)	123 (56)	110 (54)	0.653	*0.16*
High	99 (23)	52 (24)	47 (23)		
Screen time (h/week)	6 (4)	6 (3)	6 (4)	0.580	
Physical activity (h/week)	5 (2)	5 (2)	5 (2)	0.414	*0.05*
Health related physical fitness:					
Speed‐agility (4 × 10 m)^a^	17.1 (2.1)	16.9 (1.9)	17.4 (2.4)	**0.005**	*0.24*
Lower body strength (cm)	71 (20)	75 (21)	67 (17)	**< 0.001**	*0.44*
Upper body strength (kg)	7.0 (2.0)	7.0 (2.0)	6.5 (2.0)	**< 0.001**	*0.27*
Cardiorespiratory fitness (laps)	18 (10)	19 (11)	17 (8)	**0.007**	*0.24*
Balance (seg)	15 (9)	14 (9)	16 (9)	**0.003**	*0.26*
Cognition					
Numerical concepts (BaDyG)	9 (3)	10 (4)	9 (3)	0.099	*0.10*
Vocabulary (BaDyG)	11 (4)	11 (4)	11 (4)	0.100	*0.01*
Inhibition (FT)	3.65 (2.03)	3.55 (2.10)	3.74 (1.96)	0.438	*0.06*
Cognitive flexibility (DCCS)	2.44 (2.03)	2.41 (2.02)	2.47 (2.04)	0.361	*0.00*
Working memory (Word Span)	5 (2)	5 (2)	5 (2)	0.372	*0.03*

*Note:* Data are shown as mean and standard deviations, except for socioeconomic status which is shown in frequencies. Values in bold indicate statistical significance at *p* ≤ 0.050. Effect sizes were calculated using Cohen's d and interpreted as small (0.20), medium (0.50), and large (0.80) and Cramer's V for SES interpreted as small (0.10), medium (0.30), and large (0.50). Values in bold indicate statistical significance at *p* ≤ 0.050.

Abbreviations: BaDyG, Differential and General Skills Battery; BMI, Body Mass Index; DCCS, Dimensional Change Card Sort; FT, Flanker Task.

^a^ Lower values indicate better performance.

Pearson and Spearman correlation coefficients among HRPF variables, cognition and confounders are shown in Table 2. Correlation coefficients are presented with 95% CIs. All HRPF components (speed‐agility, lower‐body strength, upper‐body strength, CRF, and balance) were positively associated with all cognitive domains (numerical concepts, vocabulary, inhibition, cognitive flexibility, and working memory) (*r* = 0.11–0.38; see Table 2 for 95% CIs; all *p* ≤ 0.050). Furthermore, age and screen time were positively and negatively associated, respectively, with all cognitive domains (*r* = 0.11–0.38; *p* ≤ 0.050) and (r = 0.23–0.44; *p* ≤ 0.050). Screen time was negatively associated with numerical concepts (r = −0.15; *p* ≤ 0.050), vocabulary (r = −0.18; *p* ≤ 0.050), and working memory (r = −0.18; p ≤ 0.050). SES was positively associated with numerical concepts (*r* = 0.22; *p* ≤ 0.050), vocabulary (*r* = 0.14; *p* ≤ 0.050), and working memory (r = 0.10; *p* ≤ 0.050). Body fat was only negatively associated with working memory (*r* = 0.11; *p* ≤ 0.050), and physical activity was not associated with any cognitive domain.

**TABLE 2:** Bivariate correlation coefficients between health‐related physical fitness, cognition and potential confounders for the total sample.

	Numerical concepts (BaDyG)	Vocabulary (BaDyG)	Inhibition (FT)	Cognitive flexibility (DCCS)	Working memory (Word Span)
Speed‐agility	**0.38** (0.46;0.30)	**0.30** (0.38;0.21)	**0.36** (0.44;0.28)	**0.25** (0.33;0.16)	**0.22** (0.30;0.13)
Lower body strength	**0.24** (0.15;0.32)	**0.24** (0.15;0.32)	**0.20** (0.11;0.28)	**0.15** (0.11;0.28)	**0.11** (0.02;0.20)
Upper body strength	**0.30** (0.21;0.38)	**0.26** (0.17;0.34)	**0.23** (0.14;0.31)	**0.15** (0.05;0.23)	**0.10** (0.01;0.19)
CRF	**0.31** (0.23;0.39)	**0.22** (0.13;0.31)	**0.27** (0.18;0.35)	**0.13** (0.02;0.20)	**0.15** (0.06;0.24)
Balance	**0.33** (0.24;0.41)	**0.32** (0.24;0.40)	**0.26** (0.17;0.34)	**0.24** (0.15;0.32)	**0.19** (0.10;0.28)
Body fat %	−0.04 (−0.13;0.05)	−0.09 (−0.18; 0.00)	0.01 (−0.08; 0.10)	−0.04 (−0.13; 0.05)	**−0.11** (−0.20; −0.14)
Age	**0.44** (0.35;0.50)	**0.42** (0.33;0.49)	**0.41** (0.33;0.48)	**0.33** (0.24;0.40)	**0.23** (0.14;0.32)
SES	**0.22** (0.11; 0.28)	**0.14** (0.04;0.22)	0.08 (−0.01;0.17)	0.02 (−0.15;0.04)	**0.10** (0.07;0.24)
Physical activity	0.00 (−0.01; 0.08)	−0.01 (−0.10;0.08)	−0.03 (−0.13;0.06)	0.01 (−0.08;0.10)	−0.06 (−0.15;0.04)
Screen time	**−0.15** (−0.23;‐0.05)	**−0.18** (−0.26;‐0.08)	−0.05 (−0.14;0.05)	−0.01 (−0.10;0.09)	**−0.18** (−0.26;‐0.08)

*Note:* Data expressed as Pearson's coefficients except SES that was expressed as Spearman's rho. Correlation coefficients are presented with 95% CIs. Values in bold indicate statistical significance at *p* ≤ 0.050.

Abbreviations: BaDyG, Differential and General Skills Battery; CRF, cardiorespiratory fitness; DCCS, Dimensional Change Card Sort; FT, Flanker Task; SES, socioeconomic status.

**FIGURE 1:**
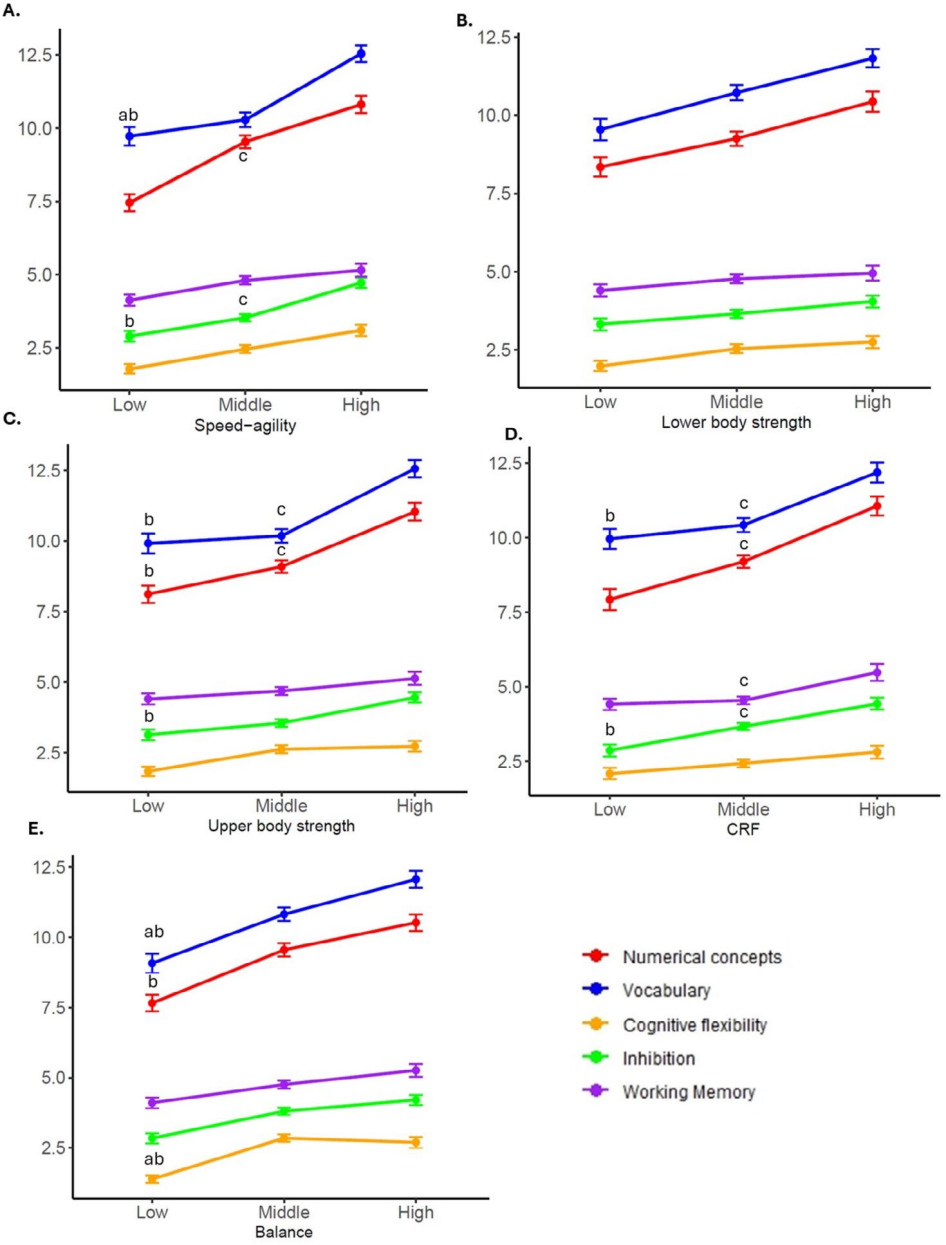
Mean difference in cognition by health‐related physical fitness categories, controlling for sex, age, socioeconomic status, and screen time. Data are shown as means and standard deviation. CRF, cardiorespiratory fitness. ^a^Differences between first and second quartiles of health‐related physical fitness components; ^b^differences between first and third quartiles of health‐related physical fitness components; ^c^differences between second and third quartiles of health‐related physical fitness components in the Bonferroni test. *p* < 0.050.

Mean difference in cognition by HRPF components, controlling for sex, age, SES, and screen time are shown in Figure 1. For speed–agility, significant group differences were found for numerical concepts (*p* < 0.001, ηp^2^ = 0.039), vocabulary (*p* = 0.003, ηp^2^ = 0.026), and inhibition (*p* < 0.001, ηp^2^ = 0.032). For upper body strength, significant differences were observed in numerical concepts (*p* < 0.001, ηp^2^ = 0.032), vocabulary (*p* < 0.001, ηp^2^ = 0.037), and inhibition (*p* = 0.042, ηp^2^ = 0.014). Regarding cardiorespiratory fitness, significant effects were found for numerical concepts (*p* < 0.001, ηp^2^ = 0.038), vocabulary (*p* = 0.009, ηp^2^ = 0.020), working memory (*p* = 0.023, ηp^2^ = 0.016), and inhibition (*p* = 0.005, ηp^2^ = 0.024). Finally, balance was significantly associated with numerical concepts (*p* = 0.001, ηp^2^ = 0.022), vocabulary (*p* = 0.004, ηp^2^ = 0.016), and cognitive flexibility (*p* < 0.001, ηp^2^ = 0.050). No significant differences were observed across lower body strength categories (all *p* > 0.05). Effect sizes for the main HRPF category effect are reported as partial eta‐squared (ηp^2^). Adjusted marginal means and Bonferroni‐corrected pairwise comparisons are presented as adjusted mean differences are provided in Table S1.

A structural equation model with two latent variables—physical fitness and cognition—was first estimated. The measurement model showed adequate fit to the data (RMSEA ≈ 0.07, SRMR = 0.07), supporting the proposed factorial structure for both constructs (Figure S1). All physical fitness indicators (upper‐ and lower‐body strength, speed/agility, CRF, and balance) and all cognitive indicators (numerical concepts, vocabulary, inhibition, cognitive flexibility, and working memory) loaded significantly on their respective latent factors (all *p* < 0.01). In this model physical fitness and cognition were strongly associated (β = 0.59, *p* < 0.001). Then, an exploratory model with sex, age, SES, and screen time as moderators showed a modest fit (Figure S2): χ^2^(57) = 361.03, *p* < 0.454; CFI = 0.95; TLI = 0.92., RMSEA = 0.042, 90% CI [0.010, 0.042], and SRMR = 0.055. Taken together, these results indicate that the model provides a reasonable approximation of the observed covariance structure and allows for substantive interpretation of the structural relations, particularly the moderation effects. In this model physical fitness was positively associated with cognition (β = 0.38, *p* < 0.001). Significant moderation effects were observed for age (β = −0.12, *p* = 0.005) and sex (β = −0.10, *p* = 0.045), indicating that the association between physical fitness and cognition was weaker at higher ages and differed by sex, being higher in boys. No significant interaction effects were found for screen time or socioeconomic status (Table S2).

**FIGURE 2:**
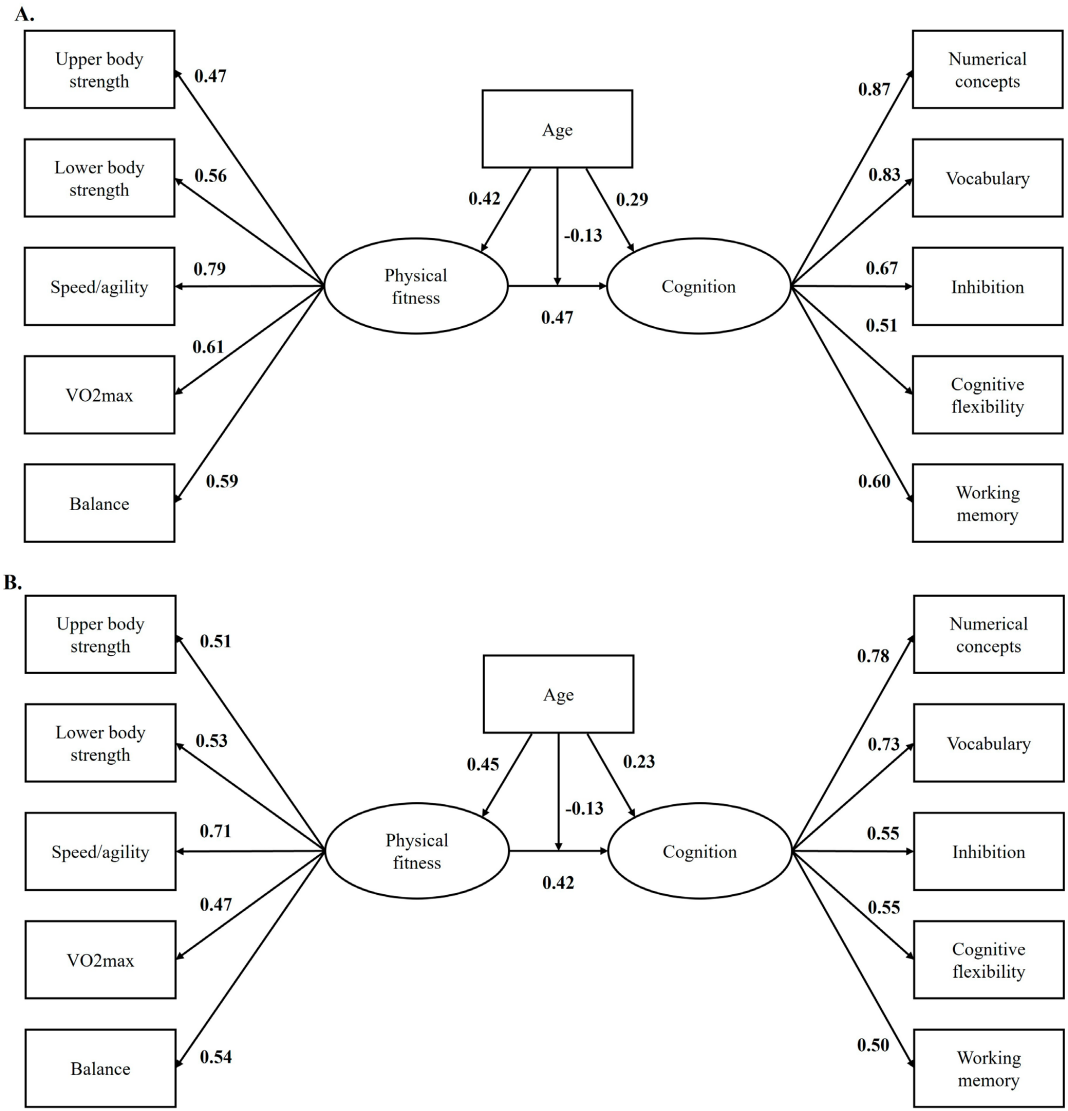
Multigroup factorial structural equation model between physical fitness and cognition moderated by age and adjusted by SES, and screen time. A Model for boys. B. Model for girls. Model fit indices: CFI = 0.975, TLI = 0.970, RMSEA = 0.041, 90% CI [0.030, 0.042], and SRMR = 0.051. Chi‐squared χ^2^(257) = 541.03, *p* = 0.001 (*N* = 458). Metric invariance: Δχ^2^(12) = 11.90, *p* = 0.454. Values in bold indicate statistical significance at *p* ≤ 0.05.

Finally, a multigroup structural equation model by sex with age as moderator adjusted by SES, and screen time (Figure 2) showed excellent overall fit to the data (χ^2^(257) = 541.03, *p* = 0.001; CFI = 0.975; TLI = 0.970; RMSEA = 0.041, 90% CI [0.030, 0.042]; SRMR = 0.039), supporting the adequacy of both the measurement and structural components of the model, with a metric invariance of Δχ^2^(12) = 11.90, *p* = 0.454, indicating equivalent loads between sex. Physical fitness was positively and significantly associated with cognition in boys and girls (β = 0.42, *p* < 0.001; β = 0.47, *p* < 0.001), respectively. Age was also directly related to cognition (β = 0.29, *p* = 0.004) and significantly moderated the association between physical fitness and cognition in both groups (β_interaction = −0.13, *p* < 0.05), indicating that the strength of this relationship decreased with increasing age.

**FIGURE 3:**
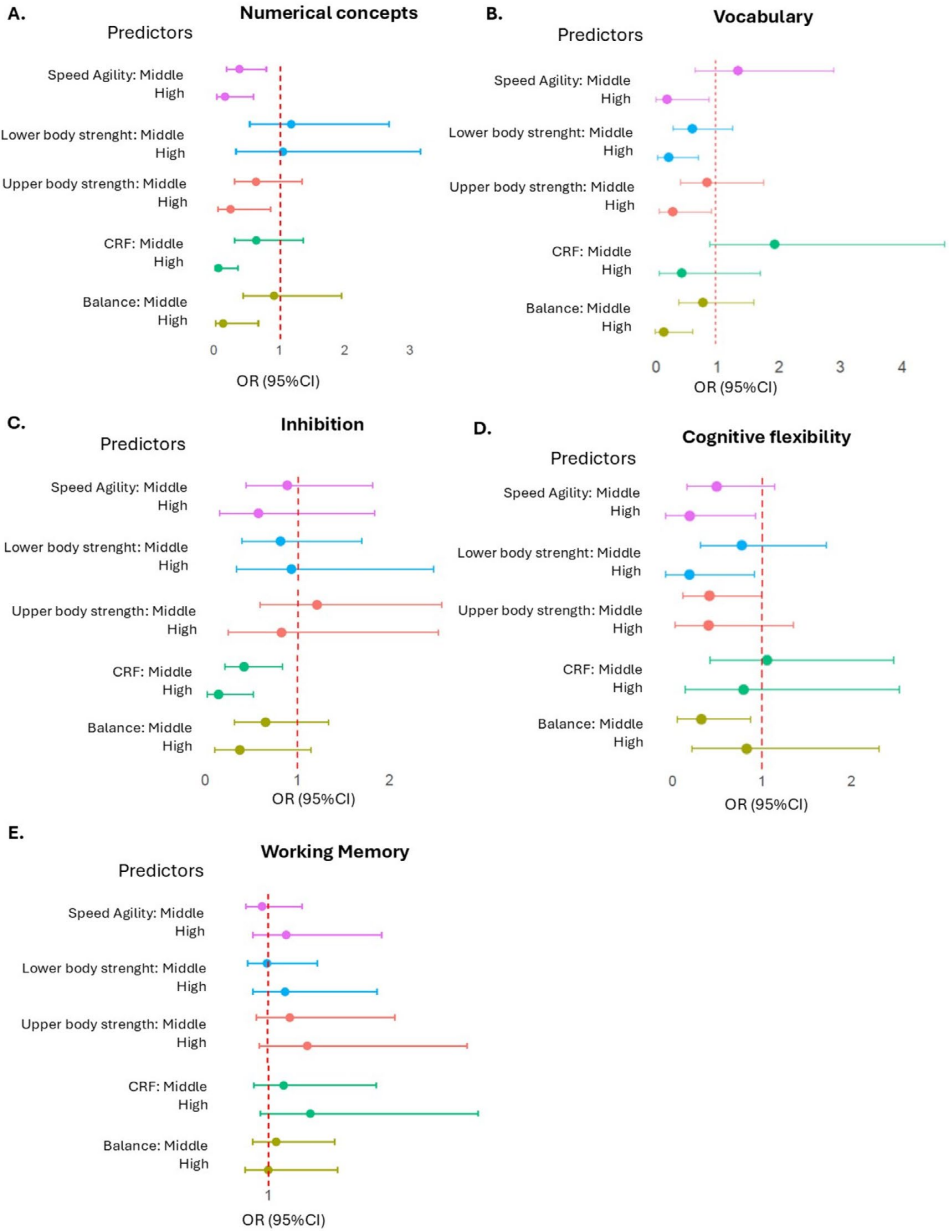
Logistic regression models predicting low cognitive achievement controlling for sex, age, socioeconomic status, and screen time. CRF, Cardiorespiratory fitness. *Low cognitive achievement was calculated as the lowest decile (*p* < 10).

Logistic regression models controlling for sex, age, SES, and screen time to estimate the odds of low cognitive achievement are presented in Figure 3. Children with middle (OR = 0.37; 95% CI: 0.18–0.80; *p* = 0.008) and high (OR = 0.16; 95% CI: 0.04–0.79; *p* = 0.024) speed‐agility, and those with high levels of upper body strength (OR = 0.26; 95% CI: 0.06–0.99; *p* = 0.015), CRF (OR = 0.05; 95% CI: 0.01–0.60; *p* = 0.036), and balance (OR = 0.13; 95% CI: 0.02–0.99; *p* = 0.050) showed significantly lower odds of poor cognitive performance. In vocabulary, a reduced odds was observed in children with high speed‐agility (OR = 0.21; 95% CI: 0.04–0.99; *p* = 0.050), low body strength (OR = 0.24; 95% CI: 0.07–0.79; *p* = 0.018), upper body strength (OR = 0.33; 95% CI: 0.10–0.98; *p* = 0.050), and balance (OR = 0.17; 95% CI: 0.04–0.78; *p* = 0.016). For inhibition, being in the middle (OR = 0.44; 95% CI: 0.22–0.87; *p* = 0.019) or high (OR = 0.14; 95% CI: 0.03–0.61; *p* = 0.008) CRF categories was associated with a lower odd of low performance. In cognitive flexibility, children with high speed‐agility (OR = 0.33; 95% CI: 0.10–0.98; *p* = 0.050), high lower body strength (OR = 0.31; 95% CI: 0.10–0.98; *p* = 0.050), middle upper body strength (OR = 0.50; 95% CI: 0.25–0.93; *p* = 0.050), and balance (OR = 0.43; 95% CI: 0.20–0.90; *p* = 0.022) had reduced odds of low achievement. Any HRPF component predicted low achievement in working memory. The values of the OR (95% CI) are shown in the Table S3.

## 4 Discussion

The results of this study showed that, even after adjusting for confounders, preschoolers with better HRPF—except lower body strength, had significantly better scores in cognition (numerical concepts, vocabulary, inhibition, and working memory). However, cognitive flexibility was only significantly associated with balance. Importantly, in addition to component‐specific analyses, we also examined a composite cognitive factor, which yielded consistent associations with health‐related physical fitness, supporting the robustness of the overall relationship between fitness and cognition beyond the differentiation of executive subcomponents. Furthermore, our findings revealed that higher levels of speed‐agility, upper body strength, CRF, and balance were associated with a lower risk of poor cognitive performance in numerical concepts, vocabulary, inhibition, and cognitive flexibility. No significant associations were found for working memory, suggesting that the relationship between HRPF and cognition may be selective depending on the cognitive domain assessed.

Beyond domain‐specific analyses, our structural equation modeling approach provides an integrated perspective on the relationship between HRPF and cognition in early childhood, congruent with previous studies [13, 40]. The strong association observed between the latent fitness and cognitive factors supports the notion that physical fitness is related not only to specific cognitive skills but also to a broader cognitive capacity underlying early learning and school readiness. This integrative finding complements the component‐level results and suggests that HRPF may act as a general correlate of cognitive development at this developmental stage. Beyond the component‐level analyses, the multigroup structural equation models provide an integrated view of the relationship between physical fitness and cognition in preschool children. Physical fitness was positively associated with global cognitive functioning in both boys and girls, with comparable effect sizes, supporting the notion that this relationship is largely independent of sex at this developmental stage, congruently with previous evidence [18, 36]. In contrast, age emerged as a relevant moderator, such that the association between physical fitness and cognition was stronger at younger ages and weakened with increasing age. Prior studies have found that age was the only predictor of cognition [18, 36]. This pattern may reflect developmental changes in both neural plasticity and the determinants of cognitive performance during the preschool years, suggesting that early preschool years may represent a sensitive period in which physical fitness is more closely linked to cognitive development [41], highlighting the potential value of early, developmentally timed fitness‐promoting interventions.

Our findings are consistent with prior research suggesting a positive relationship between HRPF and cognitive function in early childhood, especially with inhibition and working memory [13, 42] and mixed results with cognitive flexibility [16, 42]. In line with previous studies, we observed that higher CRF was linked to better inhibition and working memory [16, 43] and speed‐agility with inhibition [18]. CRF has been linked to enhanced cerebral blood flow and neurogenesis in brain areas involved in cognition [44] and speed‐agility, which involves rapid movement adaptation and coordination [45], has been associated with greater neural efficiency and increased P3 amplitude in event‐related potentials [46], suggesting a connection between complex motor skills and cognitive control [40]. The role of muscular fitness in cognitive outcomes remains less clear. While Nieto et al. found no associations with executive function [16], Zhou et al. reported a relationship between lower body strength and inhibition [18]. In contrast, our results highlighted upper body strength as being associated with inhibition, but not lower body strength. One proposed explanation involves the endocrine role of skeletal muscle, which secretes neurotrophic factors during contraction, potentially supporting brain health and cognition [47]. Nevertheless, in this study, the association between HRPF and working memory disappeared in a logistic regression model to predict low working memory achievement controlling for the confounders. Thus, inconsistencies across studies [16, 18, 48] highlight the need for further research considering confounding variables to clarify these relationships. However, these domain‐specific findings should be interpreted with caution, as executive functions in preschool‐aged children may not yet constitute clearly separable components but rather reflect partially overlapping or unitary control processes [24, 25]. Thus, observed differences across tasks may reflect task‐specific demands rather than fully distinct executive subsystems.

Our findings show associations between HRPF components and broader cognitive abilities, such as vocabulary and numerical concepts few explored in preschoolers. Although prior work in school‐aged children found no associations between HRPF domains and conceptual and verbal skills [49], to our knowledge, no previous studies have analyzed the association between HRPF and numerical comprehension. However, results in older children have found associations between HRPF components and mathematical performance [50, 51].

Furthermore, inhibition appears to be the most sensitive executive function domain to endurance and coordination training [18]. In contrast, the absence of consistent associations with cognitive flexibility aligns with prior findings and may reflect that cognitive flexibility emerges later in development and may not yet be fully established in preschool‐aged children [52]. Interestingly, only balance showed a significant association with cognitive flexibility in our study. This finding may be explained by the cognitive demands of postural control, which requires sensory integration, anticipatory planning, and the engagement of neural structures such as the cerebellum and prefrontal cortex [53, 54]. It is plausible that balance and cognitive flexibility follow similar developmental trajectories in early childhood, reflecting underlying neural integration processes. However, this result should be interpreted with caution due to the limited reliability of the one‐leg stance test in preschoolers [17].

Our logistic regression analyses revealed that higher fitness levels in speed‐agility, upper body strength, CRF, and balance were protective factors against low cognitive achievement—particularly in numerical and verbal domains, as well as inhibition. These findings reinforce the notion that HRPF may serve as a modifiable early marker of cognitive vulnerability in young children [55].

This study provides a comprehensive examination of the associations between multiple fitness and cognitive domains in a relatively large preschool sample using validated and developmentally appropriate measures. Importantly, we incorporated vocabulary and numerical concepts alongside executive functions, broadening the cognitive scope and relevance to school readiness. Second, our analytical approach accounted for multiple confounding variables including both correlational analyses and predictive modeling, offering practical insights into which specific fitness components may serve as early markers of cognitive vulnerability. Finally, the study provides evidence to a field where data remain inconsistent—preschool populations.

Nonetheless, several limitations should be noted. First, the cross‐sectional design precludes any causal inferences between HRPF and cognitive outcomes; longitudinal and intervention studies are needed to determine the directionality. Moreover, although key confounders were controlled for, other unmeasured variables may have influenced the observed relationships. Third, generalizability may be limited as participants were recruited from a specific geographic region in Spain. Fourth, difficulties in the measurement of executive function in preschoolers may not fully capture the complexity and variability of cognition. In this line, executive function in preschoolers is known to be weakly differentiated [24, 25], which may limit the interpretation of results at the component level. Fifth, the sample size was originally determined to detect intervention effects in the trial; therefore, the present analyses should be interpreted as associations within this recruited cohort rather than population‐representative estimates. No a priori sample size calculation was performed for these secondary association analyses; however, sensitivity analyses based on the achieved sample size (*n* = 522; α = 0.05, two‐sided) indicated adequate power to detect small effects, including correlations of |r| ≥ 0.122 and ANCOVA group effects of approximately partial ηp^2^ ≈ 0.02 (three categories, adjusted for age, sex, SES, and screen time). Finally, the one‐leg stance test has limited test–retest reliability in 3–5‐year‐old children [17]. Nevertheless, it was retained because static balance is a relevant neuromotor component, the task is commonly used in preschool motor competence batteries (e.g., MABC‐2), and it has been previously linked to cognitive performance [56]. Therefore, balance‐related findings should be considered exploratory and warrant confirmation using more reliable balance assessments in future studies.

The present findings indicate that HRPF may function both as a set of specific component‐level correlates and as a broader, integrated marker of cognitive development in early childhood. The consistent associations observed for cardiorespiratory fitness, speed‐agility, upper body strength, and balance, together with the strong latent‐level relationship between physical fitness and cognition, support the potential relevance of early fitness promotion for cognitive development and school readiness. The observed age‐related variation in these associations further suggests that intervention strategies may benefit from being developmentally sensitive rather than uniform across preschool populations. Our findings highlight the importance of HRPF from early childhood for enhancing cognitive development and reducing the risk of poor cognitive performance.

## 5 Perspective

This study adds novel evidence to the growing field linking physical fitness and cognitive development in early childhood. While previous research in school‐aged children has highlighted the importance of cardiorespiratory and muscular fitness for executive functions, evidence in preschoolers has remained scarce and inconsistent. Our findings extend this knowledge by analyzing different health‐related physical fitness components, cardiorespiratory fitness, speed‐agility, upper body strength, and balance as key components associated not only with the three most studied executive functions—inhibition, working memory, and cognitive flexibility, but also with broader cognitive domains including vocabulary and numerical reasoning—essential skills associated with academic achievement. In line with prior evidence, these results suggest that early promotion of health‐related physical fitness may serve as a modifiable marker of cognitive development. The potential impact of these findings lies in reinforcing preschool physical activity policies and programs that emphasize motor diverse physical activities and physical competence as foundations for learning. Future longitudinal and intervention studies should clarify causal pathways and determine whether improving specific fitness components can yield sustained cognitive benefits across childhood.

## Author Contributions

Dr. María E. Visier‐Alfonso conceptualized the idea, carried out the statistical analyses, and drafted the initial manuscript. Dr. Mairena Sánchez‐López and Dr. Vicente Martínez‐Vizcaíno conceptualized and designed the study, designed the data collection instruments, and critically reviewed and revised the manuscript. Dr. Bruno Bizzozero‐Peroni, Dr. Ana Díez‐Fernández, and Dr. Abel Ruiz‐Hermosa coordinated and supervised data collection, collected data, and critically reviewed and revised the manuscript for important intellectual content. All authors approved the final manuscript as submitted and agree to be accountable for all aspects of the work.

## Funding

This study was funded by the Spanish Ministry of Science, Innovation, and Universities (MICIN/AEI/10.13039/501100011033; ref.PID2019‐104160RB‐I00). The sponsor has no role in the research process. The sponsor has no role in the research process. BBP is supported by a grant from the Universidad de Castilla‐La Mancha co‐financed by the European Social Fund (2024‐UNIVERS‐12849).

## Conflicts of Interest

The authors declare no conflicts of interest.

## Supporting information


**Figure S1:** Factorial loads between physical fitness and cognition raw model. *Note*. Model fit indices: CFI = 0.971, TLI = 0.962, RMSEA = 0.047, 90% CI [0.031, 0.063], and SRMR = 0.038; χ^2^(34) = 68.86, *p* = 0.001 (*N* = 458). Values in bold indicate statistical significance at *p* ≤ 0.05.

**Figure S2:** Structural equation model testing the moderating effects of age and sex on the association between physical fitness and executive functions. *Note:* Model fit indices: CFI = 0.945, TLI = 0.923, RMSEA = 0.042, 90% CI [0.010, 0.042], and SRMR = 0.055. Chi‐squared χ^2^(257) = 361.03, *p* = 0.001 (*N* = 458). Values in bold indicate statistical significance at *p* ≤ 0.05. Dotted lines indicated non‐significant paths.

**Table S1:** Mean difference in cognition by physical fitness categories, controlling for confounders (sex, age, socioeconomic status, and screen time).
**Table S2:** Main and Moderation Effects of Covariates on Executive Functions (Standardized Estimates).
**Table S3:** Logistic regression models predicting risk of low cognitive achievement, without adjustment and controlling for confounders (age, sex, socioeconomic status, and screen time).

## Data Availability

The data that support the findings of this study are available from the corresponding author upon reasonable request.

## References

[1] A. Diamond , “Chapter 19 ‐ Executive Functions,” in Neurocognitive Development: Normative Development. Handbook of Clinical Neurology, ed. A. Gallagher , C. Bulteau , D. Cohen , and J. L. Michaud (Elsevier, 2020), 225–240. 10.1016/B978-0-444-64150-2.00020-4 32958176

[2] M. M. Black , S. P. Walker , L. C. H. Fernald , et al., “Early Childhood Development Coming of Age: Science Through the Life Course,” Lancet 389 (2017): 77–90. 27717614 10.1016/S0140-6736(16)31389-7 PMC5884058

[3] A. Shokrkon and E. Nicoladis , “The Directionality of the Relationship Between Executive Functions and Language Skills: A Literature Review,” Frontiers in Psychology 13 (2022): 848696. 35928417 10.3389/fpsyg.2022.848696 PMC9343615

[4] T. K. Turesky , E. S. Escalante , M. Loh , and N. Gaab , “Longitudinal Trajectories of Brain Development From Infancy to School Age and Their Relationship With Literacy Development,” Proceedings of the National Academy of Sciences of the United States of America 122 (2025): e2414598122. 40493188 10.1073/pnas.2414598122 PMC12184337

[5] B. Daelmans , G. L. Darmstadt , J. Lombardi , et al., “Early Childhood Development: The Foundation of Sustainable Development,” Lancet 389 (2017): 9–11. 27717607 10.1016/S0140-6736(16)31659-2

[6] F. B. Ortega , C. Cadenas‐Sánchez , G. Sánchez‐Delgado , et al., “Systematic Review and Proposal of a Field‐Based Physical Fitness‐Test Battery in Preschool Children: The PREFIT Battery,” Sports Medicine 45 (2015): 533–555. 25370201 10.1007/s40279-014-0281-8

[7] J. E. Donnelly , C. H. Hillman , D. Castelli , et al., “Physical Activity, Fitness, Cognitive Function, and Academic Achievement in Children: A Systematic Review,” Medicine and Science in Sports and Exercise 48 (2016): 1197–1222. 27182986 10.1249/MSS.0000000000000901 PMC4874515

[8] T. Fühner , R. Kliegl , F. Arntz , S. Kriemler , and U. Granacher , “An Update on Secular Trends in Physical Fitness of Children and Adolescents From 1972 to 2015: A Systematic Review,” Sports Medicine 51 (2021): 303–320. 33159655 10.1007/s40279-020-01373-x PMC7846517

[9] B. Masanovic , J. Gardasevic , A. Marques , et al., “Trends in Physical Fitness Among School‐Aged Children and Adolescents: A Systematic Review,” Frontiers in Pediatrics 8 (2020): 627529. 33363072 10.3389/fped.2020.627529 PMC7759499

[10] C. D'Anna , P. Forte , and E. Pugliese , “Trends in Physical Activity and Motor Development in Young People—Decline or Improvement? A Review,” Children 11 (2024): 298. 38539333 10.3390/children11030298 PMC10969615

[11] R. Guthold , G. A. Stevens , L. M. Riley , and F. C. Bull , “Global Trends in Insufficient Physical Activity Among Adolescents: A Pooled Analysis of 298 Population‐Based Surveys With 1·6 Million Participants,” Lancet Child Adolesc Health 4 (2020): 23–35. 31761562 10.1016/S2352-4642(19)30323-2 PMC6919336

[12] C. W. St. Laurent , S. Burkart , C. Andre , and R. M. C. Spencer , “Physical Activity, Fitness, School Readiness, and Cognition in Early Childhood: A Systematic Review,” Journal of Physical Activity & Health 18 (2021): 1004–1013. 34140418 10.1123/jpah.2020-0844 PMC9297301

[13] A. Veraksa , A. Tvardovskaya , M. Gavrilova , V. Yakupova , and M. Musálek , “Associations Between Executive Functions and Physical Fitness in Preschool Children,” Frontiers in Psychology 12 (2021): 674746. 34408696 10.3389/fpsyg.2021.674746 PMC8365159

[14] M. E. Visier‐Alfonso , M. Sánchez‐López , V. Martínez‐Vizcaíno , E. Jiménez‐López , A. Redondo‐Tébar , and M. Nieto‐López , “Executive Functions Mediate the Relationship Between Cardiorespiratory Fitness and Academic Achievement in Spanish Schoolchildren Aged 8 to 11 Years,” PLoS One 15 (2020): e0231246. 32275676 10.1371/journal.pone.0231246 PMC7147757

[15] D. Lubans , J. Richards , C. Hillman , et al., “Physical Activity for Cognitive and Mental Health in Youth: A Systematic Review of Mechanisms,” Pediatrics 138 (2016): e20161642. 27542849 10.1542/peds.2016-1642

[16] M. Nieto‐López , M. Sánchez‐López , M. E. Visier‐Alfonso , V. Martínez‐Vizcaíno , E. Jiménez‐López , and C. Álvarez‐Bueno , “Relation Between Physical Fitness and Executive Function Variables in a Preschool Sample,” Pediatric Research 88 (2020): 623–628. 32000261 10.1038/s41390-020-0791-z

[17] C. Cadenas‐Sanchez , B. Martinez‐Tellez , G. Sanchez‐Delgado , et al., “Assessing Physical Fitness in Preschool Children: Feasibility, Reliability and Practical Recommendations for the PREFIT Battery,” Journal of Science and Medicine in Sport 19 (2016): 910–915. 26947061 10.1016/j.jsams.2016.02.003

[18] Z. Zhou , Y. Chen , K. Huang , et al., “Relationship Between Physical Fitness and Executive Function in Preschool Children: A Cross‐Sectional Study,” BMC Sports Science, Medicine and Rehabilitation 16 (2024): 238. 10.1186/s13102-024-01028-8 PMC11616152 39633414

[19] Mediators Between Physical Activity and Academic Achievement: A Systematic Review ‐ Visier‐Alfonso ‐ 2022 ‐ Scandinavian Journal of Medicine & Science in Sports ‐ Wiley Online Library. 10.1111/sms.14107 34837413

[20] G. Raghuveer , J. Hartz , D. R. Lubans , et al., “Cardiorespiratory Fitness in Youth: An Important Marker of Health: A Scientific Statement From the American Heart Association,” Circulation 142 (2020): e101–e118. 32686505 10.1161/CIR.0000000000000866 PMC7524041

[21] E. A. Haapala , “Cardiorespiratory Fitness and Motor Skills in Relation to Cognition and Academic Performance in Children – A Review,” Journal of Human Kinetics 36 (2013): 55–68. 23717355 10.2478/hukin-2013-0006 PMC3661895

[22] P. Jylänki , T. Mbay , A. Hakkarainen , A. Sääkslahti , and P. Aunio , “The Effects of Motor Skill and Physical Activity Interventions on Preschoolers' Cognitive and Academic Skills: A Systematic Review,” Preventive Medicine 155 (2022): 106948. 34974071 10.1016/j.ypmed.2021.106948

[23] G. D. Myer , A. D. Faigenbaum , N. M. Edwards , J. F. Clark , T. M. Best , and R. E. Sallis , “Sixty Minutes of What? A Developing Brain Perspective for Activating Children With an Integrative Exercise Approach,” (2015). 10.1136/bjsports-2014-093661 25617423

[24] M. T. Willoughby , A. C. Wylie , and M. H. Little , “Testing Longitudinal Associations Between Executive Function and Academic Achievement,” Developmental Psychology 55 (2019): 767–779. 30589340 10.1037/dev0000664

[25] J. E. Karr , C. N. Areshenkoff , P. Rast , S. M. Hofer , G. L. Iverson , and M. A. Garcia‐Barrera , “The Unity and Diversity of Executive Functions: A Systematic Review and Re‐Analysis of Latent Variable Studies,” Psychological Bulletin 144 (2018): 1147–1185. 30080055 10.1037/bul0000160 PMC6197939

[26] P. Shi and X. Feng , “Motor Skills and Cognitive Benefits in Children and Adolescents: Relationship, Mechanism and Perspectives,” Frontiers in Psychology 13 (2022): 13. 10.3389/fpsyg.2022.1017825 PMC9721199 36478944

[27] C. Cadenas‐Sanchez , F. Alcantara‐Moral , G. Sanchez‐Delgado , et al., “Assessment of Cardiorespiratory Fitness in Preschool Children: Adaptation of the 20 Metres Shuttle Run Test,” Nutricion Hospitalaria 30 (2014): 1333–1343. 25433116 10.3305/nh.2014.30.6.7859

[28] BADyG i Renovado , “Manual Técnico—Editorial CEPE,” https://editorialcepe.es/.

[29] S. Weintraub , P. J. Bauer , P. D. Zelazo , et al., “I. NIH Toolbox Cognition Battery (CB): Introduction and Pediatric Data,” Monographs of the Society for Research in Child Development 78 (2013): 1–149. 10.1111/mono.12031 PMC3954750 23952199

[30] P. D. Zelazo and P. J. Bauer , National Institutes of Health Toolbox Cognition Battery (NIH Toolbox CB): Validation for Children Between 3 and 15 Years (Wiley Hoboken, NJ, 2013). 10.1111/mono.12044 23952209

[31] R. S. Fox , J. J. Manly , J. Slotkin , J. Devin Peipert , and R. C. Gershon , “Reliability and Validity of the Spanish‐Language Version of the NIH Toolbox,” Assessment 28 (2021): 457–471. 32264689 10.1177/1073191120913943 PMC7541574

[32] S. Weintraub , P. J. Bauer , P. D. Zelazo , et al., “I. NIH Toolbox Cognition Battery (CB): Introduction and Pediatric Data: NIH Toolbox Cognition Battery (CB),” Monographs of the Society for Research in Child Development 78 (2013): 1–15. 10.1111/mono.12031 PMC3954750 23952199

[33] P. D. Zelazo , J. E. Anderson , J. Richler , K. Wallner‐Allen , J. L. Beaumont , and S. Weintraub , “Ii. NIH Toolbox Cognition Battery (CB): Measuring Executive Function and Attention: NIH Toolbox Cognition Battery (CB),” Monographs of the Society for Research in Child Development 78 (2013): 16–33. 23952200 10.1111/mono.12032

[34] Executive Functioning Deficits in Relation to Symptoms of ADHD and/or ODD in Preschool Children ‐ Thorell ‐ 2006 ‐ Infant and Child Development ‐ Wiley Online Library.

[35] D. Wechsler , Wechsler Intelligence Scale for Children (Psychological Corporation, 1991).

[36] M. Nieto , B. Motos , B. Navarro , et al., Relation Between Nighttime Sleep Duration and Executive Functioning in a Nonclinical Sample of Preschool Children. 10.1111/sjop.12801 35286756

[37] M. Nieto , L. Ros , J. J. Ricarte , and J. M. Latorre , “The Role of Executive Functions in Accessing Specific Autobiographical Memories in 3‐ to 6‐ Year‐Olds,” Early Childhood Research Quarterly 43 (2018): 23–32.

[38] S. E. Gathercole , S. J. Pickering , B. Ambridge , and H. Wearing , “The Structure of Working Memory From 4 to 15 Years of Age,” Developmental Psychology 40 (2004): 177–190. 14979759 10.1037/0012-1649.40.2.177

[39] Una Propuesta de Medida de la Clase Social,” Atencion Primaria 25 (2000): 350–363. 10853507

[40] K. Wick , S. Kriemler , and U. Granacher , “Associations Between Measures of Physical Fitness and Cognitive Performance in Preschool Children,” BMC Sports Science, Medicine and Rehabilitation 14 (2022): 80. 10.1186/s13102-022-00470-w PMC9063064 35501890

[41] J. R. Best , “Effects of Physical Activity on Children's Executive Function: Contributions of Experimental Research on Aerobic Exercise,” Developmental Review 30 (2010): 331–551. 21818169 10.1016/j.dr.2010.08.001 PMC3147174

[42] Y. García‐Alonso , R. Ramírez‐Vélez , G. Legarra‐Gorgoñon , M. Izquierdo , and A. M. Alonso‐Martínez , “Associations Between Physical Fitness, Physical Activity, Sedentary Behavior and Executive Function in Preschoolers,” Pediatric Research 98 (2025): 1–8. 40000853 10.1038/s41390-025-03946-w

[43] X. Luo , F. Herold , S. Ludyga , et al., “Association of Physical Activity and Fitness With Executive Function Among Preschoolers,” International Journal of Clinical and Health Psychology 23 (2023): 100400. 37663042 10.1016/j.ijchp.2023.100400 PMC10469079

[44] C. H. Hillman , K. I. Erickson , and A. F. Kramer , “Be Smart, Exercise Your Heart: Exercise Effects on Brain and Cognition,” Nature Reviews. Neuroscience 9 (2008): 58–65. 18094706 10.1038/nrn2298

[45] Z. Liang , C. Deng , D. Li , W. L. A. Lo , Q. Yu , and Z. Chen , “The Effects of the Home‐Based Exercise During COVID‐19 School Closure on the Physical Fitness of Preschool Children in China,” Frontiers in Pediatrics 10 (2022): 10. 10.3389/fped.2022.932734 PMC9469900 36110116

[46] J. Mora‐Gonzalez , I. Esteban‐Cornejo , P. Solis‐Urra , et al., “Fitness, Physical Activity, Sedentary Time, Inhibitory Control, and Neuroelectric Activity in Children With Overweight or Obesity: The ActiveBrains Project,” Psychophysiology 57 (2020): e13579. 32249933 10.1111/psyp.13579

[47] B. K. Pedersen and M. A. Febbraio , “Muscles, Exercise and Obesity: Skeletal Muscle as a Secretory Organ,” Nature Reviews Endocrinology 8 (2012): 457–465. 10.1038/nrendo.2012.49 22473333

[48] C. Malambo , A. Klepačová , K. Brodská , C. C. T. Clark , and M. Musálek , “Relationship Among Some Coordinative and Dynamic Strength Capabilities and Constructive and Conceptual Thinking Among Preschool‐Age Children,” Frontiers in Psychology 15 (2024): 1349884. 38550648 10.3389/fpsyg.2024.1349884 PMC10973122

[49] K. Reisberg , E. M. Riso , and J. Jürimäe , “Preschool Physical Activity and Fitness Predicts Conceptual, Verbal and Perceptual Skills at School,” Journal of Sports Sciences 39 (2021): 1988–1995. 33825616 10.1080/02640414.2021.1912451

[50] V. Sember , G. Jurak , G. Starc , and S. A. Morrison , “Can Primary School Mathematics Performance be Predicted by Longitudinal Changes in Physical Fitness and Activity Indicators?,” Frontiers in Psychology 13 (2022): 796838. 35211065 10.3389/fpsyg.2022.796838 PMC8860831

[51] P. Flores , E. Coelho , I. Mourão‐Carvalhal , and P. Forte , “Relationships Between Math Skills, Motor Skills, Physical Activity, and Obesity in Typically Developing Preschool Children,” Behavioral Science 13 (2023): 1000. 10.3390/bs13121000 PMC10740894 38131856

[52] J. R. Best , P. H. Miller , and L. L. Jones , “Executive Functions After Age 5: Changes and Correlates,” Developmental Review 29 (2009): 180–200. 20161467 10.1016/j.dr.2009.05.002 PMC2792574

[53] P. Zhang , L. Duan , Y. Ou , et al., “The Cerebellum and Cognitive Neural Networks,” Frontiers in Human Neuroscience 17 (2023): 1197459. 37576472 10.3389/fnhum.2023.1197459 PMC10416251

[54] L. E. Quiñones‐Camacho , F. A. Fishburn , M. C. Camacho , L. S. Wakschlag , and S. B. Perlman , “Cognitive Flexibility‐Related Prefrontal Activation in Preschoolers: A Biological Approach to Temperamental Effortful Control,” Developmental Cognitive Neuroscience 38 (2019): 100651. 31154272 10.1016/j.dcn.2019.100651 PMC6969345

[55] K. Reisberg , E. M. Riso , and J. Jürimäe , “Physical Activity, Fitness, and Cognitive Performance of Estonian First‐Grade Schoolchildren According Their MVPA Level in Kindergarten: A Longitudinal Study,” International Journal of Environmental Research and Public Health 18 (2021): 7576. 34300027 10.3390/ijerph18147576 PMC8305144

[56] A. Frick and W. Möhring , “A Matter of Balance: Motor Control Is Related to Children's Spatial and Proportional Reasoning Skills,” Frontiers in Psychology 6 (2016): 2049. 26793157 10.3389/fpsyg.2015.02049 PMC4709580

