# Supplementary figures and images for: Association Between Health‐Related Physical Fitness and Cognition in Preschoolers: MOVI‐HIIT Study
Source: Scand J Med Sci Sports. 2026 Mar 26;36(4):e70268. doi: 10.1111/sms.70268 (PMC13022062; doi:10.1111/sms.70268)

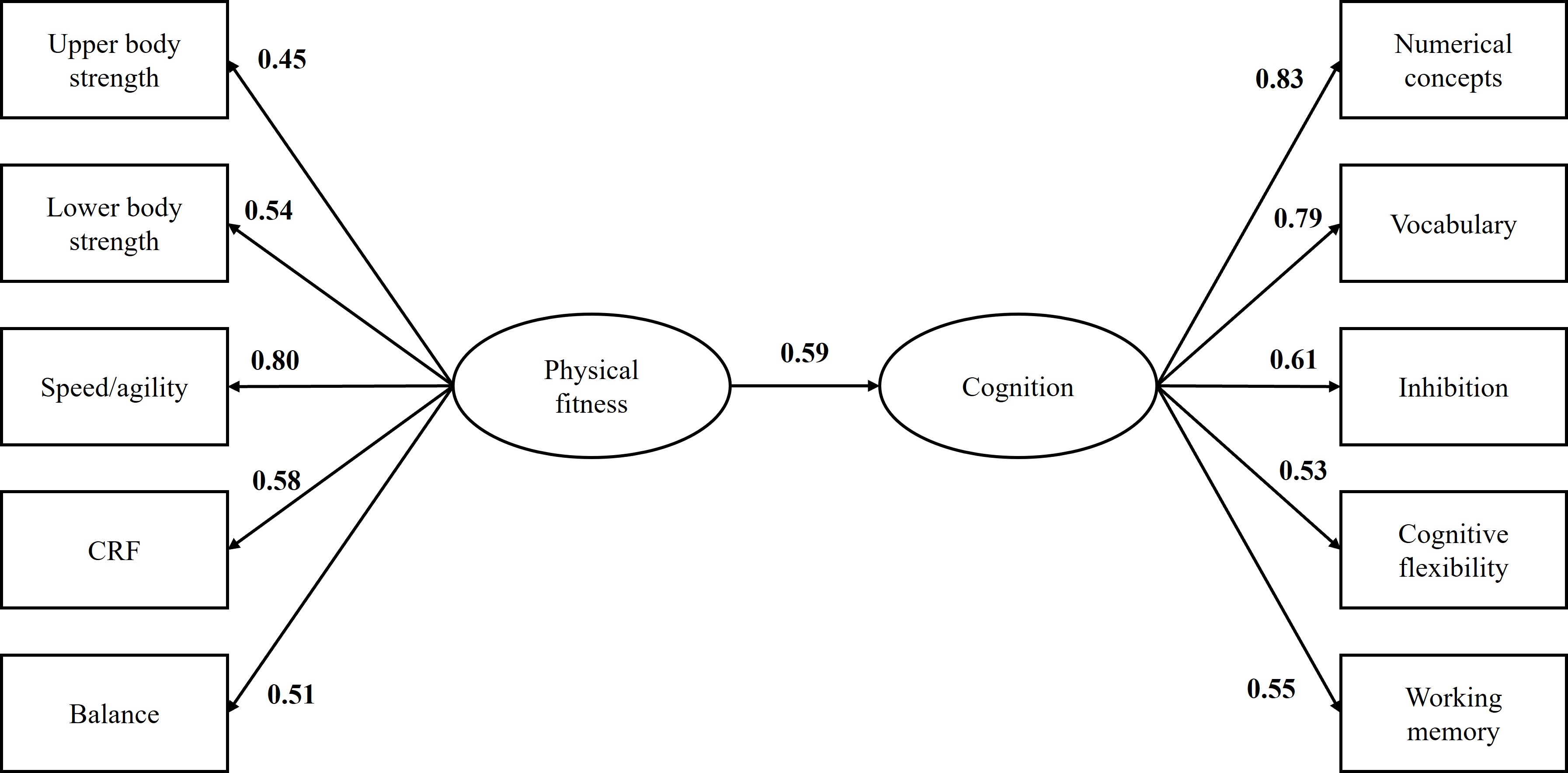

Supplement: Supplementary file 1 — Figure S1: Factorial loads between physical fitness and cognition raw model. Note. Model fit indices: CFI = 0.971, TLI = 0.962, RMSEA = 0.047, 90% CI [0.031, 0.063], and SRMR = 0.038; χ2(34) = 68.86, p = 0.001 (N = 458). Values in bold indicate statistical significance at p ≤ 0.05. [file SMS-36-e70268-s001.jpg]

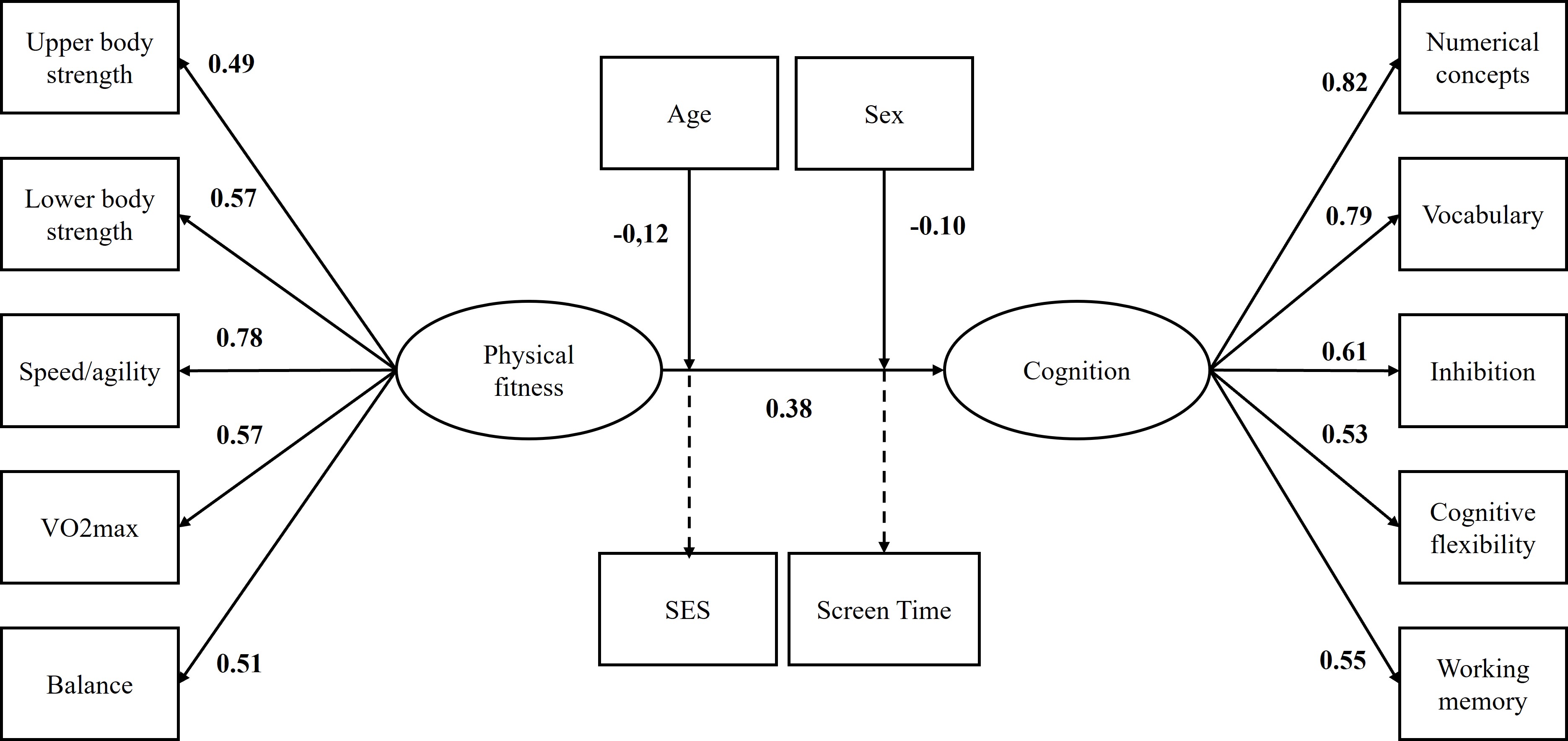

Supplement: Supplementary file 2 — Figure S2: Structural equation model testing the moderating effects of age and sex on the association between physical fitness and executive functions. Note: Model fit indices: CFI = 0.945, TLI = 0.923, RMSEA = 0.042, 90% CI [0.010, 0.042], and SRMR = 0.055. Chi‐squared χ2(257) = 361.03, p = 0.001 (N = 458). Values in bold indicate statistical significance at p ≤ 0.05. Dotted lines indicated non‐significant paths. [file SMS-36-e70268-s003.jpg]
