# Supplementary material for: Association Between Health‐Related Physical Fitness and Cognition in Preschoolers: MOVI‐HIIT Study
Source: Scand J Med Sci Sports. 2026 Mar 26;36(4):e70268. doi: 10.1111/sms.70268 (PMC13022062; doi:10.1111/sms.70268)
Supplement: Supplementary file 3 — Table S1: Mean difference in cognition by physical fitness categories, controlling for confounders (sex, age, socioeconomic status, and screen time). Table S2: Main and Moderation Effects of Covariates on Executive Functions (Standardized Estimates). Table S3: Logistic regression models predicting risk of low cognitive achievement, without adjustment and controlling for confounders (age, sex, socioeconomic status, and screen time). [file SMS-36-e70268-s002.docx]

**Table Supplementary 1.** Mean difference in cognition by physical fitness categories, controlling for confounders (sex, age, socioeconomic status, and screen time).

|  | **Physical fitness category^*^** | | | |  |
| --- | --- | --- | --- | --- | --- |
| **Speed/Agility** | **Low (Q1)**  **n = 120** | **Middle (Q2-Q3) n=246** | **High (Q4)**  **n=122** | ***p*** | ***ηp²*** |
| Numerical concepts (BaDyG) | 7.45 (3.08)^ab^ | 9.53 (3.42) | 10.80 (3.16) | **<0.001** | 0.039 |
| Vocabulary (BaDyG) | 9.72 (3.40) | 10.28 (3.78)^c^ | 12.54 (3.03) | **0.003** | 0.026 |
| Inhibition (FT) | 2.90 (1.92)^b^ | 3.53 (1.95)^c^ | 4.72 (1.90) | **<0.001** | 0.032 |
| Cognitive flexibility (DCCS) | 1.78 (1.74) | 2.45 (2.07) | 3.10 (2.05) | 0.086 | 0.011 |
| Working memory (Word span) | 4.13 (2.01) | 4.80 (2.23) | 5.15 (2.40) | 0.286 | 0.005 |
| **Low body strength** |  |  |  |  |  |
| Numerical concepts (BaDyG) | 8.34 (3.38) | 9.25 (3.41) | 10.43 (3.44) | 0.414 | 0.003 |
| Vocabulary (BaDyG) | 9.54 (3.76) | 10.72 (3.69) | 11.82 (3.18) | 0.162 | 0.008 |
| Inhibition (FT) | 3.31 (2.10) | 3.65 (2.00) | 4.05 (1.99) | 0.945 | 0.000 |
| Cognitive flexibility (DCCS) | 1.98 (1.86) | 2.54 (2.06) | 2.75 (2.03) | 0.525 | 0.003 |
| Working memory (Word span) | 4.40 (2.14) | 4.77 (2.11) | 4.95 (2.56) | 0.955 | 0.000 |
| **Upper body strength** |  |  |  |  |  |
| Numerical concepts (BaDyG) | 8.11 (3.28)^b^ | 9.09 (3.35)^c^ | 11.04 (3.32) | **<0.001** | 0.032 |
| Vocabulary (BaDyG) | 9.91 (3.71)^b^ | 10.18 (3.56)^c^ | 12.56 (3.21) | **<0.001** | 0.037 |
| Inhibition (FT) | 3.13 (2.03)^b^ | 3.55 (2.01) | 4.45 (1.88) | **0.042** | 0.014 |
| Cognitive flexibility (DCCS) | 1.83 (1.80) | 2.62 (2.10) | 2.72 (1.96) | 0.053 | 0.013 |
| Working memory (Word span) | 4.40 (2.09) | 4.68 (2.20) | 5.12 (2.43) | 0.746 | 0.001 |
| **Cardiorespiratory fitness** |  |  |  |  |  |
| Numerical concepts (BaDyG) | 7.93 (3.52)^b^ | 9.20 (3.34)^c^ | 11.06 (3.13) | **<0.001** | 0.038 |
| Vocabulary (BaDyG) | 10.96 (3.35) | 10.42 (3.78)^c^ | 12.19 (3.27) | **0.009** | 0.020 |
| Inhibition (FT) | 2.88 (1.97)^b^ | 3.68 (1.99)^c^ | 4.44 (1.96) | **0.005** | 0.024 |
| Cognitive flexibility (DCCS) | 2.10 (1.89) | 2.44 (2.02) | 2.82 (2.11) | 0.736 | 0.001 |
| Working memory (Word span) | 4.42 (1.85) | 4.55 (2.13)^c^ | 5.49 (2.70) | **0.023** | 0.016 |
| **Balance** |  |  |  |  |  |
| Numerical concepts (BaDyG) | 7.66 (3.07)^ab^ | 9.55 (3.54) | 10.53 (3.15) | **0.001** | 0.022 |
| Vocabulary (BaDyG) | 9.08 (3.62)^b^ | 10.82 (3.60) | 12.07 (3.22) | **0.004** | 0.016 |
| Inhibition (FT) | 2.84 (1.96) | 3.81 (1.99) | 4.21 (1.98) | 0.093 | 0.009 |
| Cognitive flexibility (DCCS) | 1.39 (1.48)^ab^ | 2.85 (2.03) | 2.70 (2.12) | **<0.001** | 0.050 |
| Working memory (Word span) | 4.11 (1.99) | 4.76 (2.21) | 5.26 (2.41) | 0.085 | 0.006 |

*Note.* Data are shown as adjusted marginal means ± standard error (SE). Effect sizes are reported as partial eta squared (ηp²). BaDyG = Differential and General Skills Battery; FT= Flanker Task; DCCS= Dimensional Change Card Sort.

^a^Differences between first and second quartiles of physical fitness components; ^b^differences between first and third quartiles of physical fitness components; ^c^differences between second and third quartiles of physical fitness components in the Bonferroni multiple comparisons post-hoc test.

Values in bold indicate statistical significance at p ≤ 0.050.

**Table Supplementary 2**. Main and Moderation Effects of Covariates on Executive Functions (Standardized Estimates)

| **Moderator variable** | **Main effect, β [IC]** | **P value** | **Interaction**  **CF×Moderator β (IC)** | **P value** |
| --- | --- | --- | --- | --- |
| Age (months) | 0.31 [0.037, 0.094] | <0.001 | **−0.124 [−0.280, −0.049]** | 0.005 |
| Screen time | **−0.162 [−0.097, −0.030]** | <0.001 | −0.041 [−0.122, 0.097] | 0.360 |
| Sex (0-1) | 0.091 [−0.244, -0.006] | 0.038 | **−0.093 [−0.251, −0.003]** | 0.039 |
| SES (Ref 1) | 0.048 [−0.219, 0.087] | 0.354 | −0.048 [−0.308, 0.117] | 0.401 |
| SES (Ref 1) | −0.024 [−0.125, 0.191] | 0.081 | 0.024 [−0.153, 0.304] | 0.683 |

*Note.* Values are standardized regression coefficients (β; Std.all) from the structural model predicting Executive Functions. χ²(257) = 361.03, p < .001, CFI = .923; TLI = .923 RMSEA = .042; SRMR = 0.055.

Values in bold indicate statistical significance at p ≤ 0.050.

**Table Supplementary 3.** Logistic regression models predicting risk of low cognitive achievement, without adjustment and controlling for confounders (age, sex, socioeconomic status, and screen time)

|  | | **Model 1**  **(without adjustment)** |  | **Model 2**  **(with adjustment)** |
| --- | --- | --- | --- | --- |
| Low Numerical concepts (BaDyG) | | **Odds Ratio [95% CI Lower-95% CI Upper]** |  | **Odds Ratio [95% CI Lower-95% CI Upper]** |
| Speed-agility (Ref Low) †: | Middle | **0.30 [0.15-0.59]** |  | **0.37 [0.18-0.80]** |
|  | High | **0.08 [0.02-0.33]** |  | **0.16 [0.04-0.79]** |
| Lower body strength (Ref Low): | Middle | 0.80 [0.38-1.67] |  | 1.09 [0.48-2.45] |
|  | High | 0.44 [0.16-1.19] |  | 1.04 [0.33- 3.23] |
| Upper body strength (Ref Low): | Middle | 0.52 [0.26-1.04] |  | 0.65 [0.31-1.39] |
|  | High | **0.16 [0.05-0.55]** |  | **0.26 [0.06-0.99]** |
| CRF (Ref Low): | Middle | **0.46 [0.23-0.92]** |  | 0.59 [0.27-1.26] |
|  | High | **0.05 [0.01-0.41]** |  | **0.05 [0.00-0.60]** |
| Balance (Ref Low): | Middle | 0.57 [0.29-1.14] |  | 0.93 [0.44-2.00] |
|  | High | **0.05 [0.01-0.39]** |  | **0.13 [0.02-0.99]** |
| Low Vocabulary (BaDyG) | |  |  |  |
| Speed/agility (Ref Low): | Middle | 1.04 [0.53-2.06] |  | 1.43 [0.69-2.99] |
|  | High | **0.13 [0.03-0.59]** |  | **0.21 [0.04-0.99]** |
| Lower body strength (Ref Low): | Middle | **0.51 [0.26-0.98]** |  | 0.61 [0.30-1.22] |
|  | High | **0.17 [0.06-0.53]** |  | **0.24 [0.07-0.78]** |
| Upper body strength (Ref Low): | Middle | 0.76 [0.39-1.48] |  | 0.89 [0.44-1.81] |
|  | High | **0.23 [0.07-0.70]** |  | **0.33 [0.10-0.98]** |
| CRF (Ref Low): | Middle | 1.40 [0.64-3.04] |  | 1.99 [0.86-2.56] |
|  | High | 0.31 [0.08-1.19] |  | 0.48 [0.12-2.00] |
| Balance (Ref Low): | Middle | 0.58 [0.30-1.12] |  | 0.83 [0.41-1.67] |
|  | High | **0.09 [0.02-0.39]** |  | **0.17 [0.04-0.78]** |
| Low inhibition score | |  |  |  |
| Speed-agility (Ref Low): | Middle | 0.56 [0.29-1.08] |  | 0.88 [0.43-1.81] |
|  | High | **0.19 [0.06-0.56]** |  | 0.54 [0.16-1.89] |
| Low body strength (Ref Low): | Middle | 0.55 [0.20-1.08] |  | 0.83 [0.40-1.73] |
|  | High | **0.41 [0.17-0.98]** |  | 0.90 [0.33-2.45] |
| Upper body strength (Ref Low): | Middle | 0.81 [0.41-1.61] |  | 1.17 [0.56-2.44] |
|  | High | 0.37 [0.14-1.00] |  | 0.76 [0.24-2.43] |
| CRF (Ref Low): | Middle | **0.30 [0.15-0.57]** |  | **0.44 [0.22-0.87]** |
|  | High | **0.11 [0.03-0.38]** |  | **0.14 [0.03-0.61]** |
| Balance (Ref Low): | Middle | **0.40 [0.21-0.77]** |  | 0.64 [0.31-1.32] |
|  | High | **0.15 [0.05-0.46]** |  | 0.35 [0.11-1.19] |
| Low flexibility | |  |  |  |
| Speed-agility (Ref Low): | Middle | **0.43 [0.22-0.81]** |  | 0.58 [0.30-1.16] |
|  | High | **0.16 [0.05-0.47]** |  | **0.33 [0.10-0.98]** |
| Lower body strength (Ref Low): | Middle | 0.63 [0.33-1.20] |  | 0.79 [0.39-1.56] |
|  | High | **0.18 [0.06-0.56]** |  | **0.31 [0.10-0.98]** |
| Upper body strength (Ref Low): | Middle | **0.40 [0.21-0.79]** |  | **0.50 [0.25-0.93]** |
|  | High | **0.30 [0.12-0.73]** |  | 0.49 [0.18-1.34] |
| CRF (Ref Low): | Middle | 0.74 [0.36-1.49] |  | 1.08 [0.51-2.28] |
|  | High | 0.50 [0.19-1.32] |  | 0.86 [0.30-2.45] |
| Balance (Ref Low): | Middle | **0.29 [0.15-0.59]** |  | **0.43 [0.20-0.90]** |
|  | High | **0.40 [0.18-0.89]** |  | 0.85 [0.34-1.85] |
| Low Working Memory score | |  |  |  |
| Speed/agility (Ref Low): | Middle | 0.68 [0.29-1.58] |  | 0.83 [0.34-2.02] |
|  | High | 1.13 [0.46-2.78] |  | 1.53 [0.53-2.37] |
| Low body strength (Ref Low): | Middle | 0.81 [0.34-1.93] |  | 0.96 [0.39-2.35] |
|  | High | 1.38 [0.56-3.42] |  | 1.49 [0.55-2.46] |
| Upper body strength (Ref Low): | Middle | 1.54 [0.60-3.99] |  | 1.60 [0.60-2.24] |
|  | High | 1.96 [0.70-5.49] |  | 2.01 [0.67-2.06] |
| CRF (Ref Low): | Middle | 1.18 [0.45-3.04] |  | 1.53 [0.57-2.11] |
|  | High | 1.74 [0.61-5.00] |  | 2.72 [0.72-2.79] |
| Balance (Ref Low): | Middle | 0.94 [0.42-2.09] |  | 1.17 [0.50-2.77] |
|  | High | 0.58 [0.20-1.65] |  | 0.92 [0.29-2.89] |

*Note.* CRF = Cardiorespiratory fitness. BaDyG = Differential and General Skills Battery.

†Low cognitive achievement was determined by the lowest decile.

Values in bold indicate statistical significance at p ≤ 0.050.

**Supplementary figure 1.** Factorial loads between physical fitness and cognition raw model.

*Note*. Model fit indices: CFI = 0.971, TLI = 0.962, RMSEA = 0.047, 90% CI [0.031, 0.063], and SRMR = 0.038; χ²(34) = 68.86, p = .001 (N = 458).

Values in bold indicate statistical significance at p ≤ 0.05.

**Supplementary figure 2.** Structural equation model testing the moderating effects of age and sex on the association between physical fitness and executive functions.

*Note:* Model fit indices: CFI = 0.945, TLI = 0.923, RMSEA = 0.042, 90% CI [0.010, 0.042], and SRMR = 0.055. Chi-square χ²(257) = 361.03, p = .001 (N = 458). Values in bold indicate statistical significance at p ≤ 0.05. Dotted lines indicated non-significant paths.
